# Modulation of the prostaglandin-endoperoxide synthase 2 gene expression by variant haplotypes: influence of the 3′-untranslated region

**DOI:** 10.1590/1414-431X20176546

**Published:** 2017-11-30

**Authors:** D.N. Piranda, R.B.V. Abreu, D.R. Freitas-Alves, M.A. de Carvalho, R. Vianna-Jorge

**Affiliations:** 1 Coordenação de Pesquisa, Instituto Nacional de Câncer, Rio de Janeiro, RJ, Brasil; 2 Programa de Pós-Graduação em Farmacologia e Química Medicinal, Instituto de Ciências Biomédicas, Universidade Federal do Rio de Janeiro, Rio de Janeiro, RJ, Brasil; 3 Programa de Pós-Graduação em Saúde Pública e Meio Ambiente, Escola Nacional de Saúde Pública Sérgio Arouca, FIOCRUZ, Rio de Janeiro, RJ, Brasil; 4 Instituto Federal do Rio de Janeiro, Rio de Janeiro, RJ, Brasil; 5 Programa de Pesquisa em Farmacologia e Inflamação, Instituto de Ciências Biomédicas, Universidade Federal do Rio de Janeiro, Rio de Janeiro, RJ, Brasil

**Keywords:** COX-2, PTGS2, Haplotypes, Gene expression, MCF-7, HEK293FT

## Abstract

The inducible inflammatory enzyme cycloxigenase-2 is up-regulated in cancer, and favors tumor progression. Cycloxigenase-2 is encoded by the prostaglandin-endoperoxide synthase 2 *(PTGS2)* gene, which presents sequence variations in the promoter region (PR) and in the 3′-untranslated region (3′-UTR). Different PR (rs689465, rs689466, rs20417) and 3′-UTR (rs5275) variants were generated by site-directed mutagenesis, and combined in haplotypes to access expression levels using a reporter system (luciferase) in human cells (MCF-7 and HEK293FT). Luciferase activity did not differ significantly among *PTGS2* PR constructs, except for pAAC (containing variant allele rs20417 *C*), with 40% less activity than pAAG (wild-type sequence) in MCF-7 cells (P<0.01). Despite the lack of individual significant differences, *PTGS2* PR constructs enclosing rs689466 *G* (pAGG and pAGC) showed an approximate two-fold increase in luciferase activity when compared to those containing rs689466 *A* (pAAG, pGAC, pAAC and pGAG) in both cell lines (P<0.001 for MCF-7 and P=0.03 for HEK293FT). The effect of *PTGS2* 3′-UTR sequences varied between MCF-7 and HEK293FT: MCF-7 cells showed significant reduction (40–60%) in luciferase activity (at least P<0.01), whereas HEK293FT cells showed more diverse results, with an average 2-fold increase when combined constructs (PR and 3′-UTR) were compared to respective parental PR sequences. The contribution of 3′-UTR variant (rs5275) was not consistent in either cell line. Despite the modulation of the 3′-UTR, with variable effects of rs5275, the enhancing transcriptional effect of rs689466 *G* was still detectable (P<0.0001 in MCF-7 or P=0.03 in HEK293FT cells).

## Introduction

Cyclooxygenase (COX) mediates the conversion of arachidonic acid into prostaglandin H2, the precursor of prostaglandins and thromboxanes. Two isoforms of COX are known: COX-1 is constitutively expressed, participating of physiological processes, whereas COX-2 is not detected in most resting cells, but can be induced by growth factors, cytokines, and proinflammatory stimuli (1). COX-2 catalyzes the synthesis of prostaglandin E2, which promotes cell proliferation, inhibition of apoptosis and angiogenesis (2), contributing to the pathological process of chronic inflammatory diseases and carcinogenesis (1,2).

Although up-regulated COX-2 contributes to tumor development (2), the extent of COX-2 distribution vary among individual studies (3,4). For instance, in invasive breast carcinoma, the reported frequencies of COX-2 detection by immunohistochemistry range from 17% to 84%, with a pooled estimate of 42% (3).

COX-2 is encoded by the prostaglandin endoperoxidase synthase 2 gene *(PTGS2)*, whose expression is modulated by transcriptional and post-transcriptional mechanisms (1). The promoter region (PR) of the *PTGS2* gene comprises several potential regulatory elements (1), whereas the 3′-untranslated region (3′-UTR) encloses 22–23 copies of the element “ATTTA”, which generate consensus binding sequences for proteins that regulate the stability (5) or degradation of mRNA (6). *PTGS2* is also highly polymorphic, with several single nucleotide polymorphisms (SNPs) in its regulatory regions, four of which (rs689465, rs689466, rs20417, and rs5275) appear to be the most common, with estimated global frequencies >0.1 (7).

Agúndez et al. (7) analyzed the SNPs upstream of *PTGS2* for their impact on modifying transcription factor binding sites. The authors proposed that rs689466 and rs20417 are likely to be highly relevant, since they disrupt binding sequences for MYB and E2F, respectively. In agreement, such SNPs have been described in the PR, affecting gene transcription in *in vitro* gene reporter assays (8–10). In addition to the impact of PR SNPs in gene transcription, the 3′-UTR SNP rs5275 has been shown to increase the stability of COX-2 mRNA, favoring gene expression (11).

Although *PTGS2* SNPs in the PR or in the 3′-UTR have been studied separately regarding their effects on gene transcription (8–10) or mRNA stability (11), there is no work showing their combined influence on *PTGS2* expression. Here, we developed an *in vitro* model to explore the modulation of *PTGS2* expression by haplotypes combining SNPs from both the PR and the 3′-UTR. The model was tested in two different human cell lines: MCF-7, an estrogen responsive cell line derived from metastatic human breast adenocarcinoma, which is known to express *PTGS2* (12), and HEK293FT, a highly transfectable clonal isolate derived from human embryonal kidney cells HEK293, which does not constitutively express *PTGS2* (13).

## Material and Methods

### Constructs

In order to analyze the most common *PTGS2* haplotypes, different PR and 3′-UTR variants were generated by site-directed mutagenesis using overlap extension PCR (14). Platinum Taq DNA Polymerase High Fidelity (Thermo Fisher Scientific, USA) was used in all PCR routines. Primers′ sequences for PCR and site-directed mutagenesis routines are available upon request.

#### PR constructs

Dr. Dongxin Lin (10) kindly provided *PTGS2* PR constructs in pGL3-Basic vector enclosing rs689465, rs689466 and rs20417 in the following combinations: pAAG, pAAC, pAGC, and pAGG. Two novel constructs were generated (pGAC and pGAG), using plasmids pAAC or pAAG as initial templates. PCR products were cloned in pGL3-Basic vector in *Nhe*I and *Hind*III restriction sites.

#### PR/3′-UTR constructs

The *PTGS2* 3′-UTR wild-type sequence corresponding to the region comprising nucleotides 7993–9636 (AY382629.1, GI: 34576917) was obtained by PCR amplification using a human genomic DNA as template. The 3′-UTR wild-type sequence was cloned in pGL3-control vector at the *Xba*I site, and then used as template to generate the rs5275 *C* variant, by site-directed mutagenesis. The 3′-UTR enclosing the rs5275 *C* variant was cloned in pGL3-control vector at the *Xba*I site.

The cassettes containing the *PTGS2* wild-type 3′-UTR (rs5275 *T*), or its variant (rs5275 *C*), were subcloned into PR constructs previously generated in *Xba*I sites, so that the regulatory regions (PR and 3′-UTR) of *PTGS2* would flank the luciferase reporter cassette. All constructs were confirmed by sequencing using the BigDye™ Terminator v3.1 Cycle Sequencing Kit (Thermo Fisher Scientific) and the ABI Prism 3130xl Genetic Analyser (Applied Biosystem, USA).

### Transcription activation assay

MCF-7 and HEK293FT cells were cultured in RPMI medium supplemented with 10% fetal bovine serum and 100 U/mL penicillin/streptomycin (all Thermo Fisher Scientific) in 5% CO_2_ at 37°C. Transient transfections were conducted using X-tremeGENE9^¯^_,_ for MCF-7 cells, or Fugene6¯ (both Roche, Germany) for HEK293FT cells, following manufacturer’s instructions. MCF-7 cells were plated (2.5×10^4^) in 96-multiwell plates, whereas HEK293FT cells were plated (1×10^5^) in 24-multiwell plates. After 24 h, cells were transfected with the reporter plasmid to be tested (0.2 µg for MCF-7 or 1 µg for HEK293FT cells) and the control plasmid pRL-SV40 (0.01 µg for MCF-7 or 0.5 µg for HEK293FT cells). Luciferase activity was determined 48 h after transfection, using the Dual-Luciferase Reporter Assay System (Promega, USA) following manufacturer’s instructions. At least 2 independent transfection experiments were performed for each set of results, comprising 12 replicates for each analyzed construct.

### Statistical analysis

The comparison of luciferase activities according to *PTGS2* haplotypes was performed with the GraphPad Prism 5.0 software (GraphPad Software, USA), using the non-parametric Mann-Whitney U-test for comparison of two groups, or the Kruskal-Wallis test with Dunn’s post-test for comparison of multiple groups. The threshold for significance was set at P<0.05.

## Results

**Figure 1.**
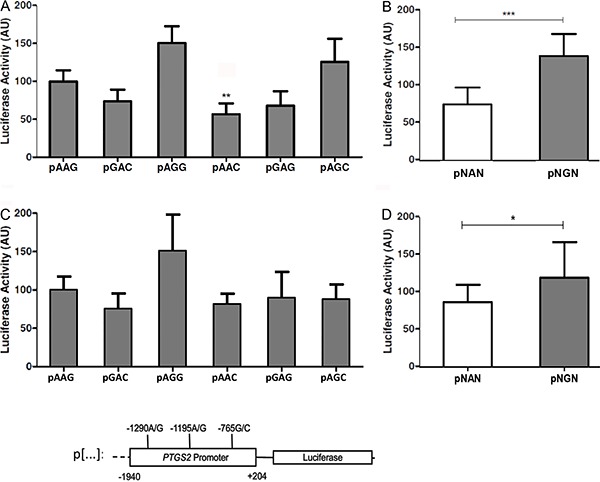
Luciferase activity of constructs containing the promoter region of the *PTGS2* gene in MCF-7 cells (*panels A* and *B*) or in HEK293FT cells *(panels C* and *D*). The results are depicted for each construct (*panels A* and *C*) or in relation to rs689466 *A/G* allele (*panels B* and *D*), combining data obtained with constructs pAAG, pGAC, pAAC and pGAG (pNAN) or with pAGG and pAGC (pNGN). Data are reported as the fold variation of luciferase activity in relation to pAAG, and are the means±SD from at least 2 independent transfection experiments, comprising 12 replicates. Statistical analyses were conducted comparing each construct to pAAG (*panels A* and *C*), or comparing pNGN *versus* pNAN (*panels B* and *D*). *P<0.05, **P<0.001, ***P<0.0001 (Mann-Whitney U-test and Kruskal-Wallis test with Dunn's post-test).

A set of six different haplotypes limited to the PR (pAAG, pGAC, pAGG, pAAC, pGAG, pAGC) was assayed in MCF-7 cells (Figure 1A and B) and in HEK293FT cells (Figure 1C and D). The statistical analysis showed no significant differences in luciferase activity, except for pAAC (containing variant allele rs20417 *C*), which presented approximately 40% reduction when compared to the wild-type sequence pAAG (Figure 1A) in MCF-7 cells. However, despite the lack of significant differences for the other constructs, the presence of the rs689466 *G* variant appears to favor gene transcription. Thus, the mean luciferase activity of pAGG and pAGC shows an approximate two-fold increase in relation to the constructs enclosing the rs689466 *A* variant (pAAG, pGAC, pAAC and pGAG), either in MCF-7 cells (Figure 1B) or in HEK293FT cells (Figure 1D).

**Figure 2.**
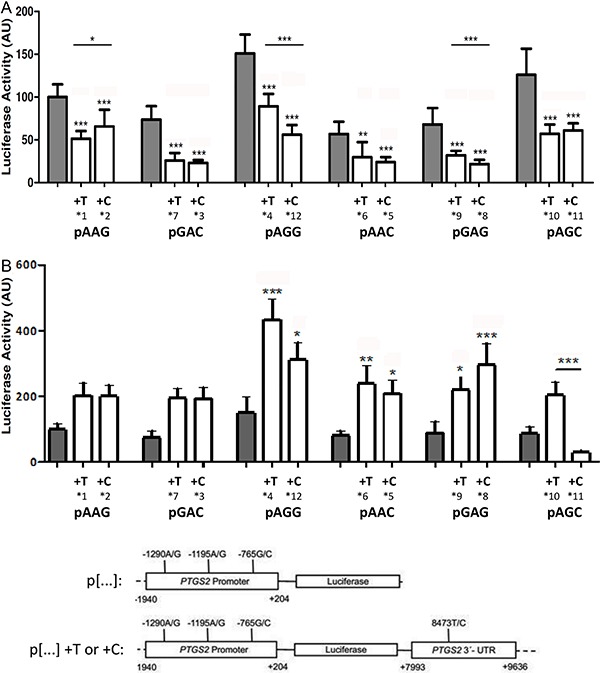
Luciferase activity of constructs containing the promoter region alone or in combination with the 3′-UTR (*rs5275 T* or *C*) of the *PTGS2* gene in MCF-7 cells (*A*) or in HEK293FT cells (*B*). Data are reported as the fold variation of luciferase activity in relation to pAAG, and are the means±SD from at least 2 independent transfection experiments, comprising 12 replicates. Cells were cotransfected with pGR-TK to normalize transfection efficiency. Statistical analyses were performed within each haplotype triad, i.e. comparing the results between constructs enclosing both PR and 3′-UTR sequences (containing either *rs5275 T* or *C*), as well as comparing each full haplotype to their respective parental PR sequence. *P<0.05, **P<0.001, ***P<0.0001 (Kruskal-Wallis test with Dunn's post-test).

Secondly, we evaluated the effect of combining the *PTGS2* 3′-UTR, containing the sequence variation of rs5275 (*T or C*), with the *PTGS2* PR variants in both cell lines (Figure 2). In MCF-7 cells, the presence of either *PTGS2* 3′-UTR sequence significantly reduced luciferase activity in relation to each respective parental PR construct (Figure 2A). In contrast, in HEK293FT cells, the presence of *PTGS2* 3′-UTR sequences led to an increase in luciferase activity (Figure 2B), except for pAGCC (haplotype *11), which showed significant reduction in luciferase activity when compared to pAGC (Figure 2B).

Concerning the effects of the 3′-UTR SNP on the combined *PTGS2* constructs, the presence of the variant allele *8473C* caused diverse results. In MCF-7 cells (Figure 2A), the variant allele led to a significantly reduced luciferase activity (P<0.01) when analyzing the related constructs: pAGGT (haplotype *4) and pAGGC (haplotype *12) or pGAGT (haplotype *9) and pGAGC (haplotype *8), but increased activity of pAAGC (haplotype *2) in comparison to pAAGT (haplotype *1) (P<0.001), and no effect when comparing the construct pairs pGACT (haplotype *7) and pGACC (haplotype *3), pAACT (haplotype *6) and pAACC (haplotype *5), and pAGCT (haplotype *10) and pAGCC (haplotype *11). In HEK293FT cells (Figure 2B), only pAGCT (haplotype *10) and pAGCC (haplotype *11) were significantly different, with the latter showing lower luciferase activity (P<0.001).

Interestingly, despite the modulation of the 3′-UTR in the final luciferase activity and regardless of the variable effects of rs5275, the enhancing transcriptional effect observed for constructs containing the rs689466 *G* variant is still detectable. Thus, the pooled luciferase activity of haplotypes containing *G* or *A* at rs689466 were 66.9±18.7 *vs* 34.3±18.2 (P<0.0001) in MCF-7 or 260.1±153.7 *vs* 219±50.7 (P=0.03) in HEK293FT cells

## Discussion

The present work is the first to evaluate the combined contribution of the four most frequent *PTGS2* SNPs on the regulation of *PTGS2* expression. Considering all possible combinations (n=16), 12 constructs were generated, comprising 10 haplotypes present in the Brazilian population (15) and 2 others that occur in populations with more diverse haploblocks (16).

The data derived from PR haplotypes indicate no individual differences in the luciferase activity (with the exception of pAAC in MCF-7 cells). However, the results suggest that the presence of the rs689466 *G* variant favors *PTGS2* transcription in relation to constructs enclosing the rs689466 *A* variant. This latter result corroborates the findings by Sakaki et al. (8) with HeLa cells, and by Pereira et al. (9) with two colon cancer cell lines (HCA-7 and HCT-116), who showed higher transcriptional activity associated with the rs689466 *G* variant. In contrast, Zhang et al. (10), also using HeLa cells, reported a 5-6-fold increase in luciferase activity driven by constructs containing rs689466 *A* when compared to those with the *G* variant at rs689466. The substitution of *A* by *G* of rs689466 apparently eliminates a MYB-binding site (7,10) and creates an E-box motif (9), which might explain the discrepancy of results observed in different cell lines. We have confirmed that both HEK293FT and MCF-7 cells were expressing the necessary elements for *PTGS2* transcription (RT-PCR approach, data not shown). Nevertheless, other relatively frequent PR SNPs, such as rs10911904, rs34984585 and rs689462, which were not studied here, might also contribute to the regulation of *PTGS2* transcription (7).

The insertion of the 3′-UTR of *PTGS2* led to different results in gene expression depending on the host cell line model. In MCF-7 cells, the presence of the *PTGS2* 3′-UTR significantly reduced luciferase activity in all haplotypes, whereas in HEK293FT cells, there was an increase, except for pAGCC, which showed significantly lower luciferase activity than all other *PTGS2* constructs. This result corroborates the notion that the *PTGS2* 3′-UTR contributes to regulate gene expression (6), and that its final effect may be either suppressive or enhancing depending on the availability of mediators, such as TTP (tristetraprolin), which favors mRNA degradation, or HuR (Hu-Antigen R), which favors mRNA stabilization (5). We have not evaluated the influence of HuR or TTP in our experiments, but a previous study by Al-Ahmadi et al. (17) indicates that MCF-7 cells over-express TTP in relation to HuR, with a TTP/HuR mRNA ratio of 2 (17). In contrast, HEK293FT cells constitutively express HuR (18), but lack TTP (19).

Concerning *PTGS2* 3′-UTR sequence variations, Moore et al. (11) suggested that rs5275 *C* variant disrupts micro-RNA-mediated mRNA degradation. Our results indicate no increase in luciferase activity with haplotypes containing rs5275 *C*, except for pAAGC (haplotype *2) in relation to pAAGT (haplotype *1) in MCF-7 cells. As the presence of micro-RNAs that may downregulate *PTGS2*, such as miR-542-3p, was not evaluated in MCF-7 or HEK293FT cells, it is possible that this post-transcriptional regulation was not fully operational in the cell models. Also, the data interpretation is limited by the fact that other SNPs in *PTGS2* 3′-UTR, such as rs36233646, which is quite common mostly in African populations (7), and rs689470, which has been reported as a putative micro-RNA binding site (20), were not evaluated in our models.

Taken together, our results corroborate the notion that the 3′-UTR of *PTGS2* has a major influence on the modulation of gene expression, with its effect depending on the cell machinery. Despite this major post-transcriptional modulation, and regardless of the variable effect of rs5275 in either cell line, the differences between *PTGS2* haplotypes appeared to remain mostly dependent on PR SNPs, especially rs689466, and its putative role on gene transcription.

We believe that the study presents an original and valid model to explore the functional impact of haplotypes affecting regulatory regions of target genes for pharmacogenetics studies, such as *PTGS2*, which may contribute to understand diverse results from observational studies, especially in complex diseases as cancer.
